# Reduced tumor growth in vivo and increased c-Abl activity in PC3 prostate cancer cells overexpressing the Shb adapter protein

**DOI:** 10.1186/1471-2407-7-161

**Published:** 2007-08-15

**Authors:** Padideh Davoodpour, Maréne Landström, Michael Welsh

**Affiliations:** 1 Department of Medical Cell Biology, Uppsala University, Uppsala, Sweden; 2 Ludwig Institute of Cancer Research, Uppsala Branch, Uppsala, Sweden; 3 Department of Genetics and Pathology, Rudbeck Laboratory, Uppsala University, Uppsala, Sweden

## Abstract

**Background:**

Induction of apoptosis is one strategy for treatment of prostate cancer. The Shb adapter protein has been found to regulate apoptosis in various cell types and consequently human prostate cancer 3 (PC3) cells were transfected to obtain cells overexpressing Shb in order to increase our understanding of the mechanisms regulating PC3 cell apoptosis.

**Methods:**

Human prostate cancer cells (PC3) were transfected with control vector or a vector containing the Shb cDNA. Clones overexpressing Shb were studied with respect to apoptosis (Dapi, M30) and c-Abl activation (Western blot for pY-245-Abl). The cells were exposed to the anti-tumor agent 2-methoxyestradiol (2-ME) and the p38 MAPK and c-Abl inhibitors SB203580 and STI-571, respectively, after which cell death was determined. In vivo tumor growth and tumor cell proliferation (Ki-67 staining) or apoptosis (active caspase 3 staining) were also determined in nude mice.

**Results:**

PC3 cells overexpressing Shb exhibited increased rates of apoptosis in the presence of the anti-tumor agent 2-ME. The Shb cells displayed increased activity of the pro-apoptotic kinase c-Abl. Pre-treatment with p38 MAPK (SB203580) or c-Abl (STI-571) inhibitors completely blocked 2-ME-induced apoptosis, implicating these two pathways in the response. The PC3-Shb cells displayed reduced tumor growth in vivo, an effect occurring as a consequence of increased apoptosis and reduced DNA synthesis.

**Conclusion:**

It is concluded that Shb promotes 2-ME-induced PC3 cell apoptosis by increased pro-apoptotic signaling via the c-Abl pathway and that this causes reduced tumor growth in vivo.

## Background

Prostate cancer is the cause of more than 1% of all deaths in men and its incidence is increasing by 2–3% per year [1]. The general prognosis for diagnosed prostate cancer remains poor and advanced prostate cancer is difficult to treat successfully. Current therapies show many side effects [2]. The endogenous estradiol-17β metabolite, 2-methoxyestradiol (2-ME) exhibits many promising signs as a candidate for future therapy of prostate cancer since treatment with 2-ME has been found to induce apoptosis in prostate cancer cells [3], while simultaneously exhibiting low toxicity to the organism. In addition, 2-ME inhibits angiogenesis [4] and reduces prostate cancer growth in vivo [3]. The growth inhibitory effects of 2-ME are associated with its effects on tubulin polymerization, leading to increased stability of microtubules and thereby cell cycle arrest [4-6]. The anti-angiogenic effects exerted by 2-ME have been linked to its inhibitory effects on hypoxia-inducible factor-1 (HIF-1) which regulates the pro-angiogenic vascular endothelial growth factor (VEGF) [7]. Interestingly, 2-ME has been reported to selectively promote cell death of human leukemia cells but not normal lymphocytes, due to its inhibitory effects on the transcription of the superoxide dismutase (SOD) enzymes [8]. Tumor cells are more dependent on SOD than normal cells, as they have higher production of superoxide radicals than normal cells. 2-ME is currently under phase I and II clinical trials.

The molecular mechanisms responsible for the cytotoxic effects of 2-ME on prostate cancer cells are only partly known, involving activation of the p38 mitogen-activated protein kinase (p38 MAPK) and the c-Jun N-terminal kinase (JNK) [3,9]. These stress kinases are important transducers of signals from the cell membrane to the nucleus and may cause apoptosis [10]. A recent study describes the involvement of Smad7 in the 2-ME apoptotic pathway as well [11]. Suppression of Smad7 contents in prostate cancer cells *in vitro *prevented the activation of p38 MAPK and subsequent apoptosis [11]. The cytotoxic response to 2-ME is thus complex and multifaceted.

Shb is an adapter protein operating downstream of certain tyrosine kinase receptors, such as the PDGF (platelet-derived growth factor) receptors and FGF (fibroblast growth factor) receptor-1 [12,13]. Shb generates protein complexes in response to activation through the assembly of signaling components via binding of these to the different domains of Shb [13,14]. Shb is pleiotropic and regulates various responses, such as apoptosis, cell migration, proliferation and differentiation [12]. Shb is a prototype for a family of adapter proteins, comprising Shb and the other members Shd, She, Shf and Shg [15,16]. All of these share a homologous SH2 domain and four conserved tyrosine phosphorylation sites. As of yet, the mechanisms by which Shb regulates cell apoptosis have remained unresolved. However, a clue as to how Shb exerts this function may come from the observation that the Shb homologue, Shd, binds c-Abl [16]. The non-receptor tyrosine kinase c-Abl is ubiquitously expressed and forms together with the Abl-related kinase (Arg) a subfamily of kinases that controls cell death under certain conditions [17,18]. The activity of c-Abl is under tight control, and binding of the c-Abl SH2 domain to its binding partners causes a conformational change that may activate the kinase [19]. Activation of c-Abl is associated with phosphorylation of regulatory tyrosine residues, such as Y245 and Y412 [20]. Several conditions of cell stress induce c-Abl activation, and consequently cell cycle arrest and cytotoxic signaling eventually causing apoptosis ensues [17,18]. We have recently described an interaction between Shb and c-Abl that regulates apoptosis [21].

To address a potential role of Shb in PC3 cell apoptosis, cells were transfected with Shb and clones overexpressing Shb obtained. Rates of apoptosis and the activation of various signaling pathways were then assessed. It is concluded that Shb sensitizes PC3 cells to apoptotic stimuli, an effect that may require augmented c-Abl activation.

## Methods

### Cell culture and reagents

Human prostate cancer cells (PC3) were obtained from ATCC and grown in cell culture medium RPMI 1640 supplemented with 10% fetal bovine serum (FBS), 2 mM L-glutamine, penicillin (100 IU/ml), in 95% air and 5% CO_2_. 2-Methoxyestradiol (2-ME), PD98059 and LY294002 were obtained from Sigma Chemical (St. Louis, MO, USA) and was dissolved in DMSO at a concentration of 10 mM. In all 2-ME experiments, DMSO alone was added to the control. SB203580 was obtained from Calbiochem (La Jolla, CA, USA). STI571 (Imatinib, mesylate or Gleevec) was obtained from Novartis and was dissolved in H_2_O at a concentration of 10 mM.

### Transfection of PC3 cells with Shb

One day after plating, PC3 cells were transfected with 3 μg Shb cDNA in pcDNA1 [22] plus 0.3 μg pcDNA3 using the Lipofectamine™ method [23] (Invitrogen, Carlsbad, CA) in 600 μl of Opti-MEM (Gibco, Grand Island, NY) for 3 hours. Clonal selection was initiated after two days by addition of 0.4 mg/ml G418. In parallel, cells were transfected with the neomycin resistance gene only (0.3 μg pcDNA3) for generating appropriate control cells. After 10–14 days, clones were isolated and expanded further. Thirty six clones transfected with the Shb cDNA were analyzed for Shb expression by Western blot analysis, and five of them were found to contain significantly elevated contents of Shb.

### Western blot analysis

One day after plating PC3 control or PC3-Shb cells (10^5 ^cells) in 3 cm dishes, these were treated with 10 μM of 2-ME for different time periods (0, 30, 60, 120, 360 min), washed with PBS and lysed directly in 150 μl of lysis buffer (2% SDS, 0.15 M Tris-HCL pH 8,8, 10% glycerol, 5% β-mercaptoethanol, bromophenolblue and 2 mM phenylmethylsulphonyl fluoride), boiled for 5 min and separated on SDS-PAGE. Proteins were electrophoretically transferred to Immobilon filters and blocked in 5% bovine serum albumin for at least one hour, after which the filters were incubated with antibodies against the following proteins: phosphorylated and total p38 (Cell Signaling, Beverly, MA) and phosphorylated and total c-Abl (Cell Signaling, Beverly, MA; Santa Cruz Biotechnology, Santa Cruz, CA). These phosphospecific antibodies recognize well-characterized activation sites of the corresponding kinases. After a brief wash with TBS + 0.1% Tween 20, membranes were probed with HRP-linked secondary antibodies (1:1000) for one hour and extensively washed. Signals were detected by an enhanced chemiluminescence (ECL) system.

### Detection of apoptosis by DAPI and M30 staining

PC3 cells (control or Shb overexpressing) were treated or not with STI-571 or with SB203580, either alone or together with 2-ME. The SB203580 (p38 inhibitor) was added to the media 1 h and STI-571 (c-Abl inhibitor) 9 h before 2-ME was administered to the cells (in the presence of the inhibitor) for 12 hours. Cells were fixed in ice-cold methanol solution for 10 minutes prior to staining for DAPI (4,6-diamidino-2-phenylindole dihydrochloride) or M30. Thereafter, the slides were stained as previously described [24]. In that study, the criteria defining apoptosis, ie nuclear pyknosis and fragmentation, are described. The number of nuclei showing morphological characteristics of apoptosis, were counted as positive in a Zeiss immunofluorescence microscope at 40× magnification. Three samples from each treatment group were evaluated. For each sample, at least 300 nuclei were counted. In order to validate the apoptotic effect of 2-ME using an independent assay, cells were stained for M30. The M30-antibody specifically recognizes caspase cleaved cytokeratin 18. The cells were stained for M30 according to the protocol provided by the manufacturer, and positive cells were counted as above.

### Detection of proliferation by Ki67-staining of tumor sections

Tumors were fixed in 10% formaldehyde and paraffin-embedded. Proliferating cells were detected on control or Shb-overexpressing tumor sections after heat-induced antigen retrieval in 0.01 M citrate buffer, pH 6.0 using the anti-rat Ki-67 antibody (MIB-5; DakoCytomation). After incubation with horseradish peroxidase-conjugated secondary antibody, immune complexes were visualized with diaminobenzidine tetrahydrochloride (DAB) precipitates, and the sections were then mounted permanently after nuclear counterstaining with Mayer's Hematoxylin. Fore each tumor, more than 250 nuclei were counted for Ki-67 positivity (dark brown nuclear stain) and the percentage calculated.

### Detection of apoptosis by caspase 3- staining of the tumor sections

Apoptosis of tumor sections was determined using an antibody against the activated form of caspase-3 (Cleaved Caspase-3 (Asp175) Ab; Cell Signaling). The cells positive for cleaved, active caspase-3 were visualized as above with DAB precipitates after incubation with horseradish peroxidase-conjugated secondary antibody. For each tumor, more than 250 cells were counted for active caspase 3 positivity (brown cellular cytoplasm) and the percentage calculated.

### In vivo tumor growth

Animal experimentation had been approved by the Uppsala Animal Ethics Committee. Control PC3 or PC3-Shb (clone 14) cells (4 × 10^6^) were inoculated subcutaneously behind the left or right forelimb on the back of nude female mice (C57Bl6 nu/nu, Taconic, Denmark) of 8 weeks of age [25,26]. Each group of cells was inoculated to ten mice (20 mice in total). The cells had been trypsinized and respuspended in 0.2 ml full culture medium prior to inoculation using a 0.6 mm needle. The appearance of tumors was carefully monitored at the time points indicated in the figure and tumor size was measured by a slide caliper and determined by the formula 0.44 × A × B^2 ^where A indicates tumor base diameter one direction and B the corresponding perpendicular value. The tumor studies were terminated by decapitation at the time points indicated in the figure or after 61 days when the tumors reached a size of 1 cm^3 ^or started to recede (PC3-Shb tumors). The tumors were rapidly taken out and fixed in 10% formaldehyde before histological analysis.

### Statistical analysis

Means ± SEM for the number of observations were determined for each experimental group. Differences were assessed using a two-tailed Students' t-test except for tumor growth, in which a parametric test (Mann-Whitney U-test) was used.

## Results

### Effects of Shb on prostate cancer PC3 cell apoptosis

**Figure 1:**
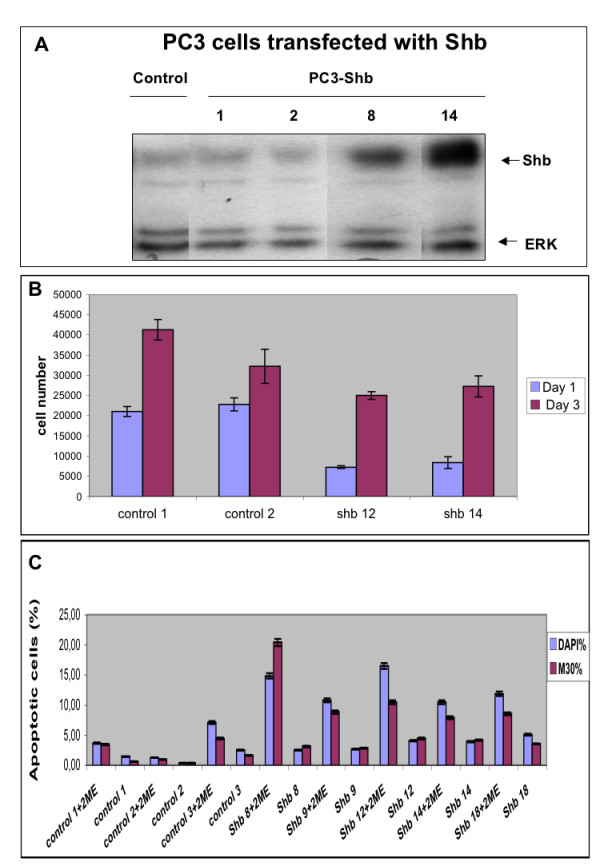
Shb protein expression in control and Shb-transfected PC3 cells (A). Different clones of PC3 cells transfected with Shb and one control-transfected clone were subjected to Western blot analysis for Shb protein as previously described [22]. Equal protein loading was assessed by ERK-protein analysis of the blot. Effect of Shb-overexpression on growth of control and Shb-overexpressing PC3 cells during 3 days (B). On day 0, cells from the different clones were trypsinized and counted in a hemocytometer, after which 10000 cells were plated in each well. Cell numbers were determined by counting in a hemocytometer on day one and three. 2-ME-induced apoptosis in control and Shb-overexpressing PC3 cells (C). Percentages of cells undergoing apoptosis as based upon DAPI nuclear staining and M30 staining were determined in three control clones and five clones overexpressing Shb after treatment (or not) with 10 μM 2-ME for 12 h. The experiments were carried out in duplicates in 3 separate experiments performed on different occasions, in which 200–600 cells from each treatment condition were counted at 40× magnification in a Zeiss immunofluorescence microscope. The mean values ± SEM are presented.

The current investigation was conducted in order to ascribe a role of Shb in prostate cancer cell apoptosis, since previous studies have implicated Shb in the regulation of apoptosis in other cell types [12]. For this purpose, PC3 prostate cancer cells were transfected with the Shb cDNA and subjected to clonal selection. Figure 1A shows Shb protein levels in one control transfected clone and four Shb transfected clones. Two of the Shb clones (clones number 8 and 14) exhibit elevated levels (two- to five-fold increase) of Shb protein expression. In addition, three additional clones (numbered 9, 12 and 18) with similarly increased Shb protein levels as that of clone 8 were found (results not shown).

Next, the basal growth characteristics of the Shb cells were tested (Figure 1B). Although equal numbers of cells were plated on day 0, the two Shb clones displayed fewer cells on day 1 than the control clones. However, all clones, both control and Shb-overexpressing, showed a similar increase in their cell numbers between day one and three. Thus, Shb overexpression does not affect the ability of PC3 cells to proliferate, although it causes these cells to become sensitized to trypsinization.

2-ME is a drug that presently is under evaluation for clinical treatment of prostate cancer due to its ability to cause prostate cancer cell apoptosis [9]. It was thus of interest to study PC3 cell apoptosis upon 2-ME treatment in the Shb-overexpressing cells (Figure 1C). Two separate assays were used for the determination of apoptosis: nuclear condensation and pyknosis determined by DAPI staining, and M30 staining for cytokeratin 18 cleavage [24,27]. Both assays detect apoptosis among adherent cells at the time of assay. All clones (both control and Shb-overexpressing) responded to 2-ME with increased rates of apoptosis. In addition, Shb-overexpression caused increased rates of apoptosis in the presence of 2-ME as compared with the three control clones. Also, it appeared as if Shb-overexpression increased apoptosis in the absence of 2-ME, although the percentage cells undergoing apoptosis was in most experiments less than 5%.

### Shb-overexpression increases c-Abl activity

**Figure 2:**
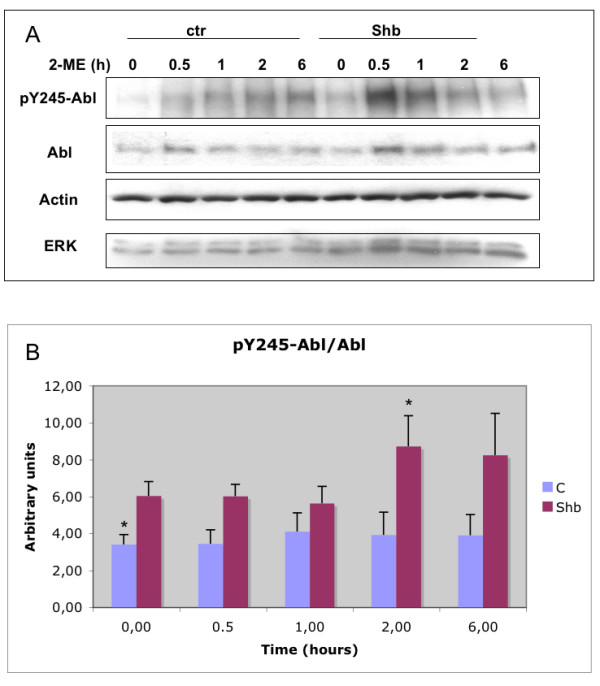
Shb-overexpression affects the phosphorylation of c-Abl in PC3 cells. Control and Shb-overexpressing PC3 cells (clone 14) were plated at equal numbers and 36 hours later treated with 10 μM of 2-ME for indicated time periods, after which all cells were lysed in SDS-sample buffer. A: Western blot analysis. The blot was probed for pY245-c-Abl and total c-Abl. The blot was also probed for beta-actin and total ERK to assess loading. B: pY-c-Abl over total c-Abl was determined by densitometry and given in arbitrary units. Means ± SEM for 4–6 separate experiments utilizing two control and five Shb-overexpressing clones are shown. * indicates p < 0.05 when compared with the PC3-Shb zero time point using a Students' t-test.

The effects of Shb on signaling pathways were investigated to find an explanation for the altered rates of apoptosis in PC3-Shb cells. The c-Abl tyrosine kinase is responsible for various apoptotic responses [17,18]. A Shb homologue, Shd [16], was found to interact with c-Abl, and we have recently reported an interaction between Shb and c-Abl that is of importance for regulation of apoptosis [21], raising the possibility that this kinase is involved in the Shb-effect. Figure 2 shows increased c-Abl activation/phosphorylation in the Shb PC3 cells without 2-ME addition. In addition, these cells responded with an additional increase of c-Abl activation in response to incubation for two hours in the presence of 2-ME that was statistically significant. Thus, the augmented apoptotic response to Shb-overexpression could involve c-Abl activation.

### Effects of p38 inhibition and c-Abl inhibition on Shb-dependent apoptosis

**Figure 3:**
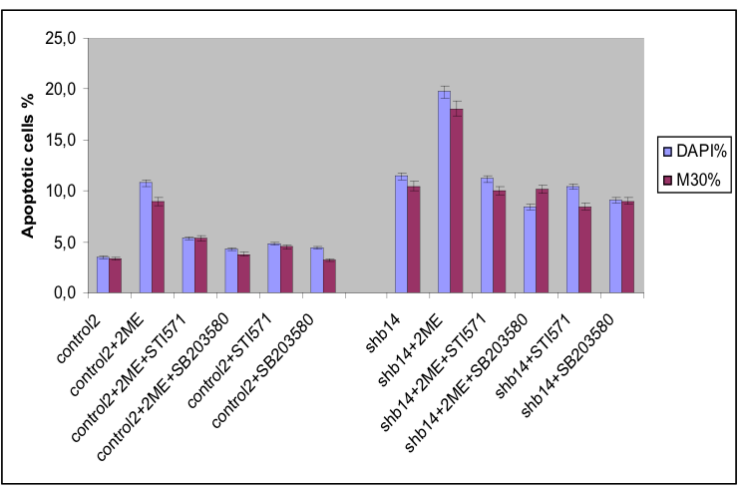
2-ME-induced apoptosis in control and Shb-overexpressing (clone 14) PC3 cells. Cells were treated or not with 10 μM of 2-ME in the absence or presence of a p38 (SB203580) or c-Abl (STI-571) inhibitor. The inhibitor was added to the media 1–9 h before initiation of 2-ME treatment. Cells were treated for 12 h, before fixation and staining with DAPI and M30 for assessing apoptosis. The experiments were carried out in duplicates in 3 separate experiments, where 200–600 cells from each treatment condition was counted at 40 × magnification in a Zeiss immuno-fluorescence microscope. Means ± SEM are shown.

To assess the contribution of c-Abl activation in Shb-dependent apoptosis in response to 2-ME, control and Shb-overexpressing PC3 cells were pre-treated with the c-Abl inhibitor STI-571 (Gleevec) prior to 2-ME exposure (Figure 3). 2-ME-induced apoptosis was completely suppressed in both the control and Shb cells. Previously, activation of the p38 MAPK has been also implicated in 2-ME-induced PC3 cell apoptosis [11]. We assessed p38 MAPK activation by Western blot analysis employing an antibody that recognizes activated p38. The results revealed that 2-ME caused p38 activation in both the control and PC3-Shb cells in a manner that was indistinguishable between these groups of cells with respect to the kinetics and magnitude of p38 stimulation (results not shown), suggesting that the effect of Shb does not involve p38 MAPK. The involvement of p38 MAPK was further examined by pre-treatment of cells with the p38 inhibitor SB203580 and studying 2-ME induced apoptosis. Again, the 2-ME response was completely suppressed in the control and Shb cells. The data suggest activation of both the p38 and c-Abl pathways as necessary for 2-ME-induced PC3 cell apoptosis.

### In vivo tumor growth

**Figure 4:**
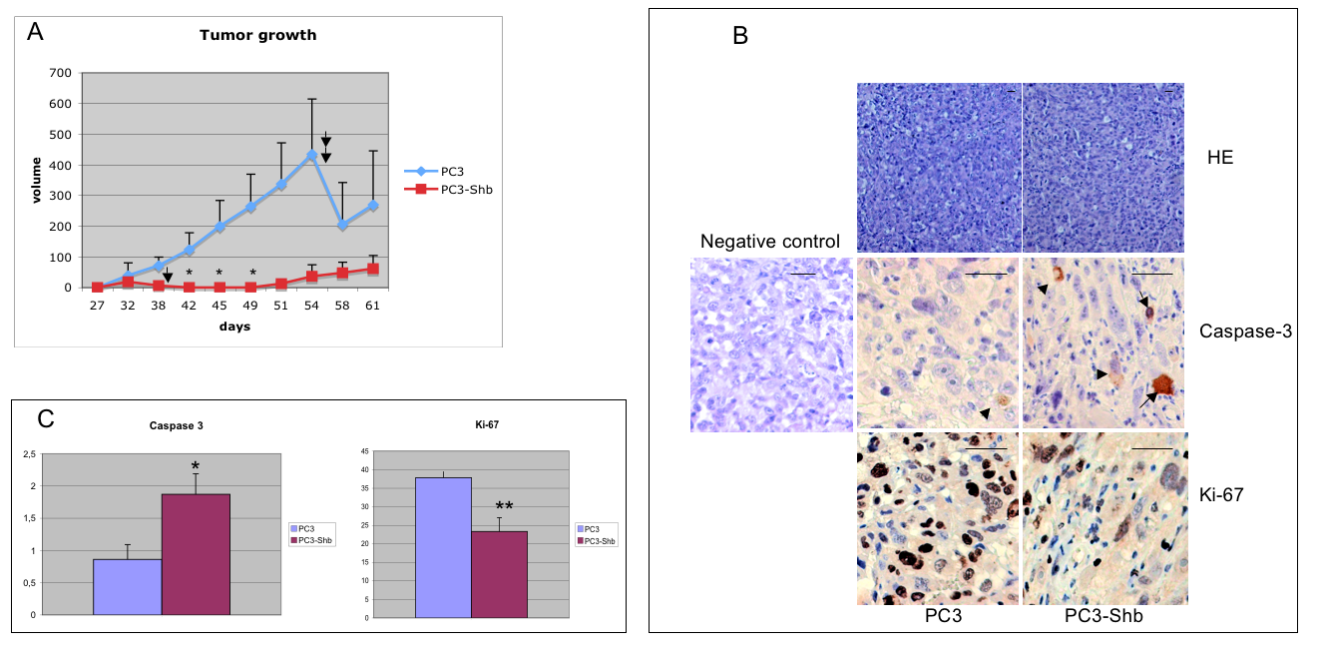
In vivo tumor growth of control or Shb-overexpressing PC3cells. A. Cells were injected subcutaneously into female nu/nu mice. Tumor growth was carefully monitored. The tumor take rate was 6/10 for the control PC3 cells and 3/10 for the Shb-overexpressing PC3 cells (clone 14). All PC3-Shb tumors regressed when they reached a size of 14, 50 and 170 mm^3^, respectively. Means tumor volume ± SEM are given. Arrows indicate sacrifice of mice due to the tumor size approaching 1 ml in the controls or in the Shb case tumor regression. * indicates p < 0.05 when tested against control using a Mann-Whitney U-test. B. PC3 control or PC3-Shb tumor cell morphology after staining for Hematoxylin-Eosin (HE), active caspase 3 or Ki-67. A negative control for active caspase 3 staining is also shown. Arrows indicate cells positive for caspase 3. Original magnification was 100× for HE and 400× for Ki-67 and caspase 3. Horizontal bars in subpanels indicate 50 μm. C. PC3 control or PC3-Shb tumor cell apoptosis or proliferation after staining for active caspase-3 or Ki-67. Percentage cells positive for active caspase-3 are given. Means ± SEM for 6 or 3 observations, respectively. * indicates p < 0.05 with a Student's t-test. Tumor cell DNA synthesis by Ki-67 staining. Percentage Ki-67 positive cells are give as in C. ** indicates p < 0.01 with a Student's t-test.

Nude mice were injected with PC3-control or PC3-Shb cells after which tumor growth was monitored [25,26]. The tumor take rate of the control cells was 6 out of 10. For the Shb cells, the tumor take rate was 3 out of 10. The control tumors expanded at a rapid rate (Figure 4). Although the Shb tumors became visually detectable at a similar time point, their subsequent growth was much slower (Figure 4). All three of the PC3-Shb tumors regressed when they reached a size of 14, 50 and 170 mm^3^, respectively. These animals were sacrificed before complete disappearance of the tumors (arrow indicates sacrifice of the mouse carrying the first tumor that regressed, the others were sacrificed after 61 days when their regression was severe but not complete). The control tumors were retrieved either when they reached a volume of 1 cm^3 ^as indicated by the arrows or at the end of the experiment. Tumor morphology, tumor cell proliferation assessed by Ki67-staining and tumor cell apoptosis visualized by staining for active caspase-3 was then determined on histological sections (Figure 4). No obvious differences in tumor histological morphology were noted between the control or Shb tumors. However, when examining rates of tumor cell apoptosis after the significant reduction in the Shb tumor sizes, the Shb tumors were found to display elevated rates of apoptosis (Figure 4). Furthermore, their rates of DNA synthesis were reduced. The data suggest that Shb-overexpression reduces PC3 cell in vivo tumor growth as a consequence of a combined effect on apoptosis and DNA synthesis.

## Discussion

As previously reported in fibroblasts [22], pancreatic islet beta-cells [28] and endothelial cells [29,30], overexpression of the SH2 domain adapter protein Shb in human prostate cancer cells (PC3) also causes increased apoptosis under certain conditions. Particularly the increased apoptotic response to 2-ME is of interest since 2-ME is currently under consideration in clinical trials as a therapy for prostate cancer. Elucidation of the mechanisms of the Shb effect in this context may provide additional insight into the action of 2-ME that could eventually bear fruit in the form of novel clinical applications.

Previous work has implicated activation of p38 MAPK and increased Smad7 as signaling events responsible for the action of 2-ME [11,31]. Although p38 activation was noted in response to 2-ME in the Shb cells, this effect was not accentuated, suggesting that Shb uses another pathway to promote 2-ME-induced apoptosis. The Smad7 content and GSK-3β kinase activity were not affected by Shb overexpression (results not shown), arguing against an involvement of these pathways in the Shb effect.

Shb activates the pro-apoptotic kinase c-Abl. We have recently observed interactions between Shb and c-Abl that have a bearing on the regulation of apoptosis [21], and our present observations are in line with this. Numerous apoptotic pathways have been reported to be activated by c-Abl, for example p73 [32], p38 MAPK [33], JNK (c-Jun N-terminal kinase) [33], as well as inactivation of NF-κB [34]. In this study, we have not explored the p73 and NF-κB pathways, but we could not detect activation of JNK in the control or Shb cells (results not shown) and p38 MAPK was activated to a similar extent in the control and Shb cells (results not shown), leaving the downstream signaling pathways of c-Abl presently operating unknown.

To verify the participation of the c-Abl pathway as responsible for Shb-dependent 2-ME-induced death, cells were treated with the c-Abl inhibitor STI-571 (Gleevec). This completely blocked 2-ME-induced death. STI-571 is not entirely specific for c-Abl, and may inhibit the PDGF receptors as well [35]. However, the presently described effect of STI-571 is not likely to involve the PDGF receptors, since PDGF reduces Shb-dependent cell death [22]. In vivo trials employing STI-571 as treatment of prostate cancer via PDGF receptor inhibition have been initiated [36]. However, anti-apoptotic effects of STI-571 via c-Abl inhibition could be a complicating factor confounding the interpretation of such in vivo studies.

The overexpression of Shb also causes reduced in vivo tumor growth of PC3 cells. This effect occurs in the absence of 2-ME treatment and is pronounced. In addition, it occurs as a consequence of a combined effect on apoptosis and cell proliferation. Again it is likely that the perturbation that Shb causes in intracellular signaling, ie activated pro-apoptotic signaling via c-Abl, will create an intracellular milieu unsupportive of tumor growth in vivo. However, the data do not exclude alternative possibilities, for example that the effects are secondary to other processes, such as reduced angiogenesis, despite the fact that we could not detect any major differences in vascularization between the control or Shb tumors.

The data suggest that both the c-Abl and p38 MAPK pathways are required for 2-ME-induced PC3 cell death, since addition of these inhibitors completely blocked the 2-ME effect. Understanding the presently described Shb-dependent augmentation of pro-apoptotic signaling via c-Abl could provide additional measures for improving prostate cancer therapy in vivo.

## Conclusion

Overexpression of the Shb adapter protein causes increased apoptosis of human prostate cancer PC3 cells in response to the anti-tumor agent 2-ME. This is likely to result from increased c-Abl activity. As a consequence of Shb overexpression, there is reduced tumor growth in vivo. Promoting Shb signaling may generate means to improve prostate cancer treatment.

## Abbreviations

Abl- Abelson kinase

GSK-3β- Glycogen synhase kinase-3β

DAPI- 4,6-diamidino2-phenylindole dihydrochloride

ERK- Extracellular signal-regulated kinase

ECL- Enhanced chemiluminescence

2-ME- 2-methoxyestradiol

MAPK- Mitogen-activated protein kinase

PC-3- Prostate cancer cells

PBS- Phosphate buffered saline

TBS- Tris-buffered saline

SDS page- SDS-Polyacrylamide gel electrophoresis.

## Competing interests

The author(s) declare that they have no competing interests.

## Authors' contributions

All three authors have contributed to the experimental design, experimentation, analysis and interpretation of data and writing of the manuscript. All authors have read and agreed on the contents of the final manuscript.

## Pre-publication history

The pre-publication history for this paper can be accessed here:


